# Do We Still Need Conventional Holter ECGs in the Era of Smart Devices?

**DOI:** 10.31083/RCM51122

**Published:** 2026-10-16

**Authors:** Reto Stump, Jonas Brügger, Patrick Badertscher

**Affiliations:** ^1^Department of Cardiology, Cardiovascular Research Institute Basel (CRIB), University Hospital Basel, University of Basel, 4031 Basel, Switzerland

**Keywords:** wearable electronic devices, telemetry, ECG ambulatory, atrial fibrillation

## Abstract

Wearable devices for rhythm monitoring are increasingly available and are being used by patients across a broad spectrum of technologies, including smartwatches with electrocardiogram (ECG)-recording functions and ECG patch systems. Early development of automated rhythm classification algorithms using artificial intelligence primarily focused on atrial fibrillation (AF), although indications and validated use cases differ substantially between device types and continue to evolve. This review aims to provide an overview of contemporary smart devices, their capabilities in arrhythmia care, and future perspectives. Currently, two main technologies are used for rhythm assessment: photoplethysmography (PPG) and single- or multi-lead electrocardiography. PPG enables continuous monitoring of heart rate and pulse regularity, whereas ECG acquisition is typically user-initiated. The use of a 30-second single-lead ECG (SL-ECG) strip with physician confirmation for the diagnosis of AF is supported by current ESC AF guidelines. Systematic screening approaches using wearables have demonstrated higher AF detection rates compared with standard diagnostic pathways; however, whether this translates into improved stroke prevention remains to be proven. Beyond AF, wearables may be particularly useful for infrequent symptomatic arrhythmias, such as supraventricular tachycardias, by enabling SL-ECG recording during symptoms and thereby supporting diagnosis and management. Additional applications include monitoring of QT prolongation, conduction disturbances, and rhythm surveillance following interventions. As an established alternative to user-dependent ECG acquisition, ECG patches provide continuous rhythm recording for up to 30 days and are generally more user-friendly than conventional Holter monitoring. A key challenge remains automated rhythm detection for non-AF arrhythmias, as well as a relevant proportion of inconclusive tracings in real-world populations, for example in the presence of conduction abnormalities or frequent premature atrial or ventricular complexes. From a physician’s perspective, clear workflows and dedicated technical solutions are essential to manage the growing volume of data, and reimbursement mechanisms for analysis of these recordings must be established. Ultimately, the future of wearable-based rhythm monitoring lies in its structured integration into clinical workflows, enabling more individualized and optimized arrhythmia care. In parallel, combining rhythm information with additional health metrics may facilitate a more holistic approach to prevention and management of cardiovascular disease. Despite all technical advances, traditional Holter ECG still has irreplaceable value in patients with frequent arrhythmia symptoms or when coronary vasospasms or Brugada syndrome are suspected.

## 1. Introduction

In 1961, Norman J. Holter [1] presented the first portable electrocardiogram (ECG) recording device that allowed the recording of an ECG-tracing in an out-of-hospital setting. More than 60 years later, there is a broad range of devices for outpatient rhythm monitoring, such as implantable loop recorders, ECG patches and smartwatches with an ECG-recording function, with evolving indications for each device. The introduction of smart devices amenable to the general population shifted the paradigm from a physician-only initiated examination to a consumer-initiated examination. Additionally, smartwatches not only enable cardiac rhythm monitoring but can also evaluate the wearer’s fitness and overall well-being, with the aim of improving health outcomes. It is projected that for smartwatches, the compound annual growth rate is 24.75% - reaching a market volume of 695 million devices sold by the year 2030 [2].

The large body of research in the field of wearables and arrhythmias has focused on the detection of atrial fibrillation (AF). Since wearables can record high-quality ECG tracings and their algorithms can reliably detect atrial fibrillation, their use for AF diagnosis was already recommended in the 2020 ESC Guidelines, provided that recordings are confirmed by manual interpretation [3]. These recommendations were enforced in the 2024 ESC AF Guidelines [4]. In contrast to the conventional Holter ECG, wearables offer the possibility to record several parameters simultaneously, potentially improving our understanding of interactions between different conditions, such as obstructive sleep apnea and AF [5].

This review aims to provide an overview of various smart devices, their capabilities for comprehensive arrhythmia care and future perspectives.

## 2. Wearables

### 2.1 What is on the Market

Wearables can assess heart rhythm using two distinct technologies: photoplethysmography (PPG) and the acquisition of single- or multi-lead electrocardiograms (SL-ECG or ML-ECG).

#### 2.1.1 Photoplethysmography

Most devices contain sensors for PPG which can continuously monitor heart rate and pulse regularity. PPG sensors detect changes in blood volume in the microvasculature, resulting in a plethysmogram, that can be assessed for regularity [6]. PPG can also be used to monitor additional parameters such as blood oxygen saturation and respiration and enables cuffless blood pressure monitoring in selected devices [7]. Moreover, it has been shown that a smartphone with a dedicated app can be used to record PPG either by placing a finger on the camera (contact) or, as shown by preliminary data, via contactless facial video [8,9].

#### 2.1.2 Electrocardiogram

In 2018, Apple introduced the first smartwatch capable of recording a SL-ECG. Since then, consumers can now choose between smartwatches from several manufacturers [10]. In contrast to the continuous analysis of the photoplethysmogram, ECG acquisition in smartwatches is typically user-initiated and requires two electrodes of the watch to be in contact with different body parts. For regular use, the watch needs to be worn on the wrist so that the electrode on the back side is in contact with the skin. Then, a finger of the other hand touches a specified part of the watch case that contains the second electrode. This results in a SL-ECG corresponding to Einthoven lead I of a regular 12-lead ECG. As an alternative to smartwatches, handheld ECG devices are available, which offer the capability of recording up to six leads [11]. Depending on the manufacturer, ECG strips of 30 to 40 seconds can be recorded which are then automatically analyzed and classified as “sinus rhythm”, “atrial fibrillation” or “inconclusive”. Examples and tracings from several smartwatches and from one handheld ECG device are displayed in Fig. 1. Furthermore, several devices offer continuous ECG monitoring such as skin patches, chest straps or T-shirts with built-in electrodes.

**Fig. 1.**
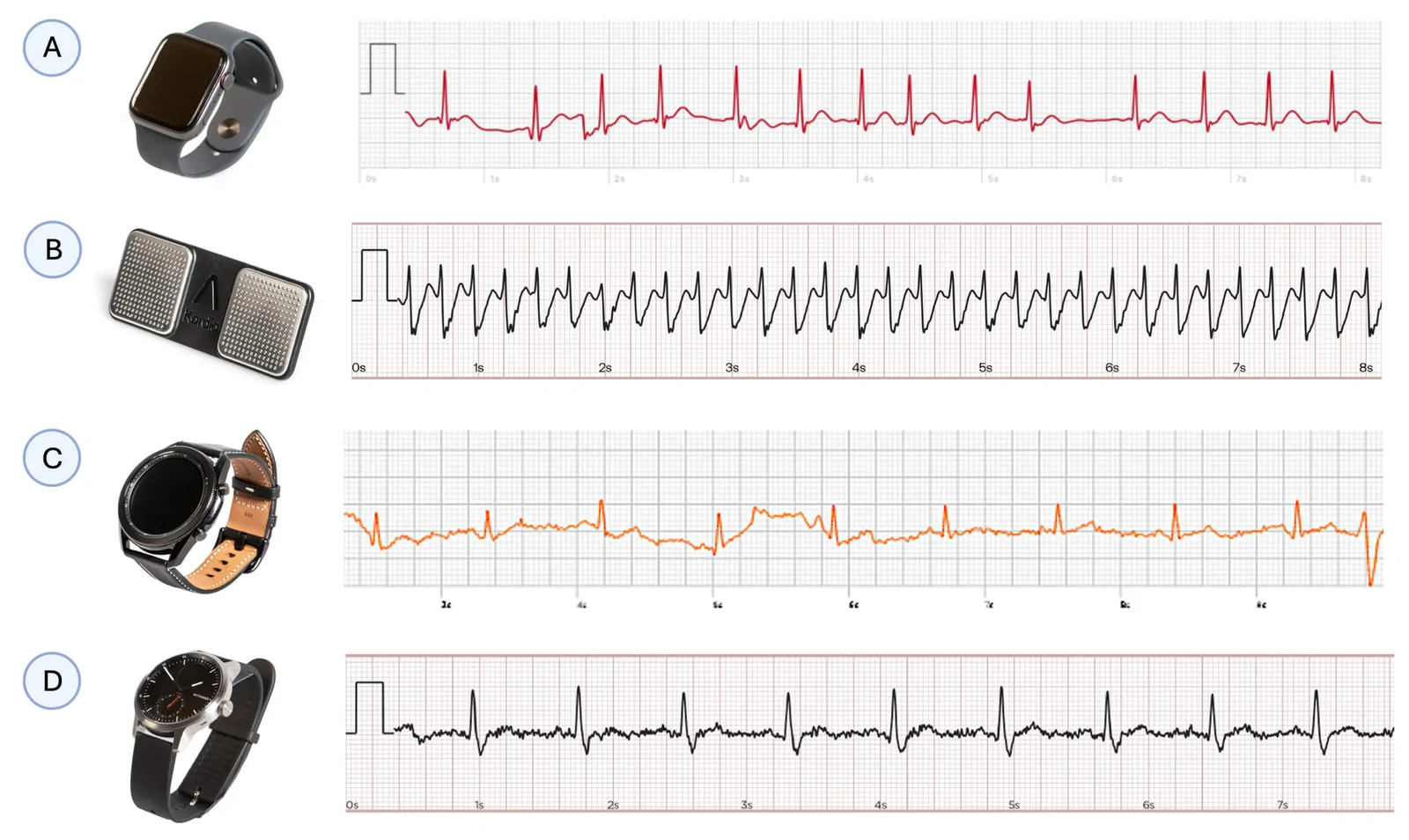
**Examples of four different devices (three smartwatches and one handheld device) displaying ECG tracings from routine clinical practice**. (A) Apple Watch 6 (Apple Inc., Cupertino, CA, USA): This tracing was recorded during an episode of palpitations. The automated analysis of the single-lead ECG (SL-ECG) suggested atrial fibrillation (AF), which was subsequently confirmed by an electrophysiologist. This enabled an early diagnosis without the need for further arrhythmia diagnostics. Following cardiology consultation, the patient was referred directly for pulmonary vein isolation. (B) AliveCor KardiaMobile (AliveCor, Mountain View, CA, USA): This tracing was recorded during a 15-minute episode of palpitations with sudden onset and termination and was transmitted digitally to the Basel Wearable Clinic (wearableclinic.ch). Although the heart rate exceeded the upper limit for automated rhythm analysis, the signal quality allowed reliable manual interpretation. The SL-ECG demonstrated a regular narrow-complex tachycardia at 248 bpm with probable retrograde P waves concealed within the QRS complex. After contacting the patient, an electrophysiological study was arranged directly, avoiding additional diagnostic appointments. (C) Galaxy Watch3 (Samsung Electronics, Suwon, Republic of Korea): This SL-ECG was recorded in the absence of cardiac symptoms and was classified as AF by the automated algorithm. Because of uncertainty regarding the result, the tracing was digitally transmitted to the Basel Wearable Clinic. Manual interpretation demonstrated sinus rhythm with a premature ventricular complex, thereby preventing unnecessary diagnostic procedures or treatment. (D) This SL-ECG was recorded by a patient with a history of syncope and known complete right bundle branch block. Despite the presence of artifacts and a widened QRS complex, the automated algorithm correctly identified sinus rhythm. AF, atrial fibrillation; bpm, beats per minute; SL-ECG, single-lead electrocardiogram.

### 2.2 Atrial Fibrillation

#### 2.2.1 Current Guideline Recommendations

According to the 2024 ESC Guidelines for the management of AF as well as the 2023 ACC/AHA/ACCP/HRS Guideline for the Diagnosis and Management of Atrial Fibrillation, a diagnosis of AF can be established with documentation on a 12‑lead ECG or a physician-reviewed SL-ECG or ML-ECG [4,12]. Wearables using PPG to monitor heart rhythm continuously can trigger an irregular rhythm notification suggesting that an ECG should be obtained in case an irregular pulse is detected. However, those notifications alone must not be regarded as diagnostic and should not be the sole basis for initiating oral anticoagulation [13].

#### 2.2.2 Smartwatch Accuracy in Detecting Atrial Fibrillation

Smartwatches are potentially useful in screening for subclinical AF aiming at starting oral anticoagulation earlier if indicated and thus reducing the risk of stroke. Large industry-sponsored studies (Apple Heart study, Fitbit Heart study and Huawei study) shared a similar study design: PPG was used to assess beat-to-beat variability and if their algorithm suspected AF, an ECG patch or a Holter ECG was performed [14,15,16]. Despite their sample size of >200,000 participants, the proportion of people aged ≥65 years ranged between 1.8% and 12.1%, which is less representative for patients at risk for AF. The study designs did not allow assessment of sensitivity and specificity, however, the positive predictive value of PPG used for AF detection in the Apple Heart Study was 84% (95% CI, 76%–92%) and 91.6% in the Huawei study (95% CI: 91.5%–91.8%).

Wearables that are able to record a SL-ECG or ML-ECG are usually equipped with dedicated algorithms for classification of the rhythm strip as “possible atrial fibrillation”, “sinus rhythm”, or “inconclusive”. A study in a real-world cohort comparing the diagnostic accuracy of five different devices showed a sensitivity of 58–85% and a specificity of 69–70% [10]. The number of inconclusive tracings ranged between 17 and 26%, however, cardiologist rhythm interpretation was possible in 95% of the inconclusive tracings. Patients with tracings automatically classified as inconclusive tend to have more baseline ECG anomalies such as low voltage, ≥2 premature atrial or ventricular beats or conduction delay [17]. The amount of inconclusive tracings tended to decrease over time most likely due to improvements in the artificial intelligence (AI) algorithms [18]. Alternatively, an analysis by a third-party device-agnostic AI algorithm is able to improve the automated SL-ECG analysis resulting in a rate of inconclusive tracings as low as 1–4% that is comparable with the reduction achieved by physician review [19].

#### 2.2.3 Stroke Prevention – Does It Work?

AF is the most common sustained cardiac arrhythmia worldwide with a lifetime risk of >30% [20]. In patients with a cryptogenic stroke prolonged rhythm monitoring detects AF in 12–14% [21,22]. Therefore, from a physician and patient perspective, using wearables for simplified AF screening, starting oral anticoagulation earlier in eligible patients and finally reducing the rate of thromboembolic strokes seems appealing but has not yet been demonstrated. Currently, there is no published trial that has assessed whether smartwatches with automated AF detection algorithms are able to reduce thromboembolic events. The yet-to-be published HEARTLINE study is designed to answer this question [23].

### 2.3 Performance of Smartwatches in the Detection of Other Arrhythmias and Cardiac Pathologies

#### 2.3.1 Typical Atrial Flutter

A smartwatch SL-ECG can aid in diagnosing typical atrial flutter. However, smartwatches are mostly worn on the left wrist, thereby recording a SL-ECG that corresponds to Einthoven lead I. An automated interpretation by the algorithm might be less reliable as the flutter waves are often rather small in that lead, resulting in a sensitivity for automated detection of only 25% [24]. This can be improved by wearing the smartwatch on the left ankle and touching the second electrode with the right hand, thereby obtaining a SL-ECG corresponding to Einthoven lead II [25].

#### 2.3.2 Paroxysmal Supraventricular Tachycardias

The three most common differential diagnoses in paroxysmal supraventricular tachycardia (PSVT) are AV-nodal reentry tachycardia, AV reentry tachycardia in the presence of an accessory pathway and focal atrial tachycardia. Patients usually present with sudden onset palpitations. Episode frequency varies from less than once a year up to daily episodes while the length ranges between less than a minute up to several hours. Therefore, it can be difficult to capture an ECG during symptoms with non-invasive monitoring. As wearables are right at hand, they offer the possibility to acquire an SL-ECG with a delay of only a few seconds after symptoms have started and can therefore be a valuable tool in the diagnosis of PSVT (Fig. 1B) [26]. Furthermore, in patients who already possess such a device, using its diagnostic functions reduces costs and can shorten the time to effective therapy. Additionally, in patients with an antegrade-conducting accessory pathway, ventricular preexcitation is visible in most cases using a standard smartwatch SL-ECG while even more cases can be detected when the watch is also placed on the precordium corresponding to ECG leads V1, V3 and V6 [27].

#### 2.3.3 QT Prolongation and Brugada Syndrome

QT prolongation (≥450 ms in men; ≥460 ms in women) can be genetic or acquired and has the potential to result in polymorphic ventricular tachycardias (Torsades de pointes). The differential diagnosis for an acquired QT prolongation is broad, including electrolyte disturbances, such as hypokalemia, hypomagnesemia or hypocalcemia, or several drugs [28]. Current guidelines recommend serial ECGs on a regular basis when drugs with QT-prolonging potential are initiated [29]. Wearables potentially simplify the surveillance by offering remote monitoring. The recently published practical compendium on antiarrhythmic drugs suggests the use of event recorders or smartwatches in patients after initiating sotalol [30]. Currently, only the Withings ScanWatch offers automatic QTc measurement from a SL-ECG which may underestimate the QTc interval [31]. Therefore, it is currently recommended to measure it manually, although this can also yield substantially variable results depending on the physician’s expertise [32]. Furthermore, it needs to be emphasized that an SL-ECG cannot capture QT dispersion across leads. This could be overcome by acquiring several SL-ECGs with a smartwatch from the standard and additional locations [27,33]. Alternatively, AliveCor’s KardiaMobile 6L hand-held device records a 6-lead ECG and is the only FDA-approved hand-held device for the measurement of the QTc interval [34].

Brugada syndrome is a channelopathy characterized by a typical ECG pattern in right precordial leads V1 or V2 positioned in the second, third or fourth intercostal space, and is associated with an increased risk of sudden cardiac death [29]. It is well established that in most patients the Brugada ECG pattern is only intermittently present [35] while the amount of time the Brugada pattern is present (“Brugada burden”) could be useful for risk stratification [36]. Therefore, prolonged ECG monitoring could be beneficial for the diagnosis and management of Brugada syndrome. It has been demonstrated that a Brugada pattern can be detected from an Apple watch SL-ECG that was acquired from the precordial lead V1 position in the fourth intercostal space [27] or from the lead V2 position in the third intercostal space [37]. However, evidence is insufficient to recommend smartwatches as a diagnostic tool for Brugada syndrome.

#### 2.3.4 Myocardial Ischemia

An SL-ECG by itself is rarely diagnostic of a myocardial infarction and there is no wearable with a built-in algorithm to interpret ST deviations. However, it is possible to acquire a 9-lead ECG by recording nine sequential ECG tracings at different locations (Einthoven I, II, III and modified precordial leads V1-6) with the Apple watch resulting in intervals and amplitudes comparable with a 12-lead ECG [38,39]. Furthermore, the amplitude of ST deviations in ST-elevation as well as in non-ST-elevation myocardial infarction are similar in a 12-lead ECG and 9-lead smartwatch ECG [40]. Smartwatch ECGs might be an alternative in situations where physical medical contact is delayed or impossible, e.g., during a flight, in the mountains or in very rural areas [39]. There are certain limitations with regards to the ECG acquisition and the further processing. According to the studies, a very high percentage of patients were able to record the different ECG tracings successfully after only brief instruction. However, we believe that this percentage would be much lower in an out-of-hospital emergency setting. Patients would need easily understandable, step-by-step instructions that guide them through the acquisition and the transmission of the tracings.

#### 2.3.5 Conduction Abnormalities and Postinterventional Rhythm Monitoring

SL-ECGs can reveal relevant conduction abnormalities such as high-degree or complete AV block as well as right or left bundle branch block [41,42], however, bundle branch blocks should always be verified with a 12-lead ECG. Therefore, recording a SL-ECG twice daily and in case of symptoms during the first 1–2 weeks after transcatheter aortic valve implantation (TAVI) was feasible and enabled earlier detection of relevant arrhythmias [43]. Furthermore, continuous heart rate monitoring by the PPG sensor of the smartwatch can facilitate the detection of sudden onset bradycardia [44]. However, underlying conduction disturbances still pose a challenge for correct rhythm classification by the device algorithm [17]. Apart from post-TAVI rhythm monitoring PPG-based or SL-ECG-based wearables can be used for remote post ablation monitoring, such as pulmonary vein isolation [45,46].

#### 2.3.6 Cardiomyopathies and Adult Congenital Heart Disease

Nasarre et al. [27] demonstrated that SL-ECGs acquired with an Apple watch from modified lead positions can identify typical ECG signs of hypertrophic cardiomyopathy (HCM) or arrhythmogenic right ventricular cardiomyopathy (ARVC). The characteristic ECG signs are visible in SL-ECGs from lead positions comparable to where these are identifiable in a 12-lead ECG (V1-V3 for ARVC, V5-V6 and I for HCM). In daily practice, as patients usually present tracings recorded from the left wrist, physicians might encounter tracings from which a HCM could be suspected if it displays high R-wave amplitude and T-wave inversions while ECG signs of an ARVC will hardly be encountered.

In patients with adult congenital heart disease (ACHD) wearables could play a role in earlier detection of arrhythmias as this patient population is at elevated risk for atrial and ventricular arrhythmia. Striepe et al. [47] assessed SL-ECGs recorded with the Apple watch in ACHD patients. Overall, 77% of the SL-ECGs were correctly analyzed by the algorithm compared to the manual interpretation of a 12-lead ECG. However, the study yields limited insights as most of the patients were in sinus rhythm [47]. The underlying structural and conduction heterogeneities as well as a higher proportion of non-AF arrhythmias in these patients are suspected to increase the number of inconclusive tracings.

### 2.4 Challenges for the Use of Wearables in Clinical Practice

Despite the growing number of people who wear smartwatches, several issues require careful discussion. From the physician’s perspective, clear workflows and dedicated technical solutions must be implemented to manage the increasing volume of data. At the same time, the responsibilities surrounding self‑initiated diagnostics, responsibilities for patient-initiated recordings, particularly outside office hours, must be clarified. Moreover, reimbursement mechanisms for the analysis of these recordings must be established.

From the patients’ side, it is important to recognize that not all devices have been formally approved as medical devices by regulatory authorities. Patients should be advised to purchase an officially approved device in order to guarantee a certain quality standard. In addition, the protection of all acquired health‑related data must be ensured, particularly when the data are stored on company‑owned cloud-based platforms. For health‑conscious individuals, self‑initiated diagnostics without medical supervision can lead to anxiety and uncertainty, especially in the case of false‑positive findings. At the University of Basel, the Basel Wearable Clinic (www.wearableclinic.ch) was developed as a device‑agnostic online platform through which patients can upload smartwatch‑derived or other SL-ECGs from devices that carry the Conformité Européenne (CE) mark or are approved by the US Food and Drug Administration (FDA) [48]. The recordings are reviewed by an arrhythmia specialist, and patients receive a report including recommendations for further management. This solution illustrates how self‑initiated diagnostics and professional care can be combined effectively.

When wearables are used for frequent rhythm monitoring over a longer period of time, patient adherence remains a limitation. Especially in cases where monitoring is not continuous and requires frequent user activation within the app [49]. Another challenge lies in the accessibility and price of these devices, which not all patients are able to afford.

## 3. ECG Patches

ECG patches offer continuous rhythm monitoring from several days up to 30 days. From the patient perspective, patches are more comfortable to wear, causing less skin irritation and are easy-to-use, which is associated with higher adherence [50]. Additionally, from the clinician perspective, it has been shown that the ZIO Patch (iRhythm Technologies, San Francisco, California) yielded more analyzable time than a traditional three-electrode Holter device [51]. Most FDA-cleared or CE-marked patches record an SL-ECG, but there are some multi-lead patches on the market. Furthermore, certain products offer real-time inpatient and outpatient arrhythmia monitoring and automated analysis by connecting the patch with a portable transmission device. Tracings from two different patches in common clinical scenarios are displayed in Fig. 2.

**Fig. 2.**
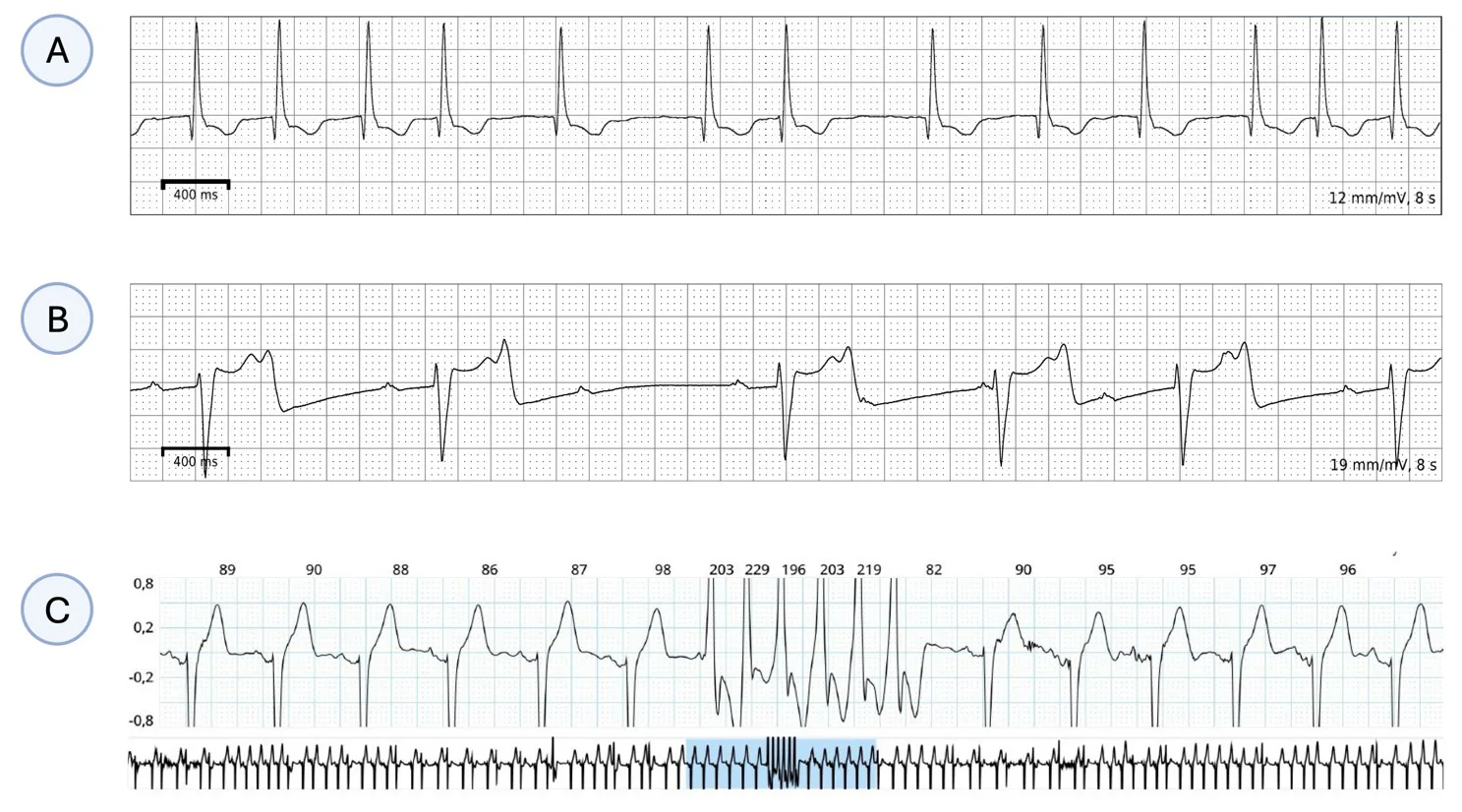
**Representative ECG tracings obtained from ECG patch monitoring**. (A) A ZIO Patch (iRhythm) was used for atrial fibrillation (AF) screening in a patient with ischemic stroke. Automated analysis correctly identified an episode of AF. (B) A ZIO Patch (iRhythm) was applied for extended ambulatory ECG monitoring following transcatheter aortic valve implantation (TAVI) and correctly detected an episode of high-grade atrioventricular (AV) block. (C) A Smartheart ECG patch (MySmartHeart AG, Zurich, Switzerland) was used for risk stratification in a patient with arrhythmic mitral valve prolapse. Although frequent ventricular ectopy and slow non-sustained ventricular tachycardia were already known, extended monitoring revealed a rapid non-sustained ventricular tachycardia (>180 bpm), resulting in implantation of an implantable cardioverter-defibrillator rather than an implantable loop recorder (ILR). AF, atrial fibrillation; bpm, beats per minute; TAVI, transcatheter aortic valve implantation; ILR, implantable loop recorder.

Apart from technical advancements of the sensors, the improvement of the algorithm’s accuracy for the automated analysis is also essential for an efficient workflow. Hannun et al. [52] trained and validated a deep learning model with 91,232 SL-ECGs from an ECG patch to classify 12 rhythm classes (sinus rhythm, noise, 10 arrhythmias). The deep neural network achieved an average area under the receiver operating curve of 0.97, which is comparable to experienced cardiologists [52].

In AF screening, the use of ECG patches in elderly people seems appealing as it can result in a higher proportion of patients receiving oral anticoagulation [53,54]. However, whether this transforms into a reduced stroke rate remains debatable. In the randomized controlled GUARD-AF trial including 11,905 patients ≥70 years, AF screening using a 14-day ECG patch did not result in a reduction of strokes compared to usual care [55]. Notably, the trial was terminated early due to the COVID-19 pandemic, and the number of ischemic events was low.

In patients who have had a stroke, ECG patches offer a simple method for prolonged rhythm monitoring with an increased AF detection rate compared to a short duration Holter ECG [56]. However, AF diagnosed by prolonged rhythm monitoring after a stroke might be a separate entity with a lower stroke risk than AF diagnosed by a 12-lead ECG [57]. The ongoing Sweet spoT for cArdiac Rhythm monitorinG After sTrokE (STARGATE) trial is designed to improve the management of these patients (NCT05717504).

In conclusion, ECG patches transform rhythm monitoring in specific situations and enable patient-centered, high quality arrhythmia care. Despite the increasing use, their impact on the healthcare system needs to be evaluated.

## 4. Implantable Loop Recorders

For long-term surveillance, implantable loop recorders (ILR) offer continuous rhythm monitoring over several years. They are the preferred strategy for rarely occurring and unforeseeable events which cannot be captured with non-invasive devices. A meta‑analysis of randomized studies, published in the 2018 ESC guidelines on syncope, compellingly demonstrated the superiority of the ILR strategy. The pooled diagnostic yield was 46% in the ILR group versus only 12% in the control groups using conventional diagnostics, corresponding to a relative likelihood of 3.6 (95% CI: 2.4–5.3; *p* = 0.001). Notably, the effect remained consistent across the various studies, regardless of the specific control strategy employed. Accordingly, ILR received a class I, level A, recommendation in high-risk patients after exclusion of structural heart disease [58]. An example of an ILR recording in a patient with recurrent syncope is shown in Fig. 3.

**Fig. 3.**
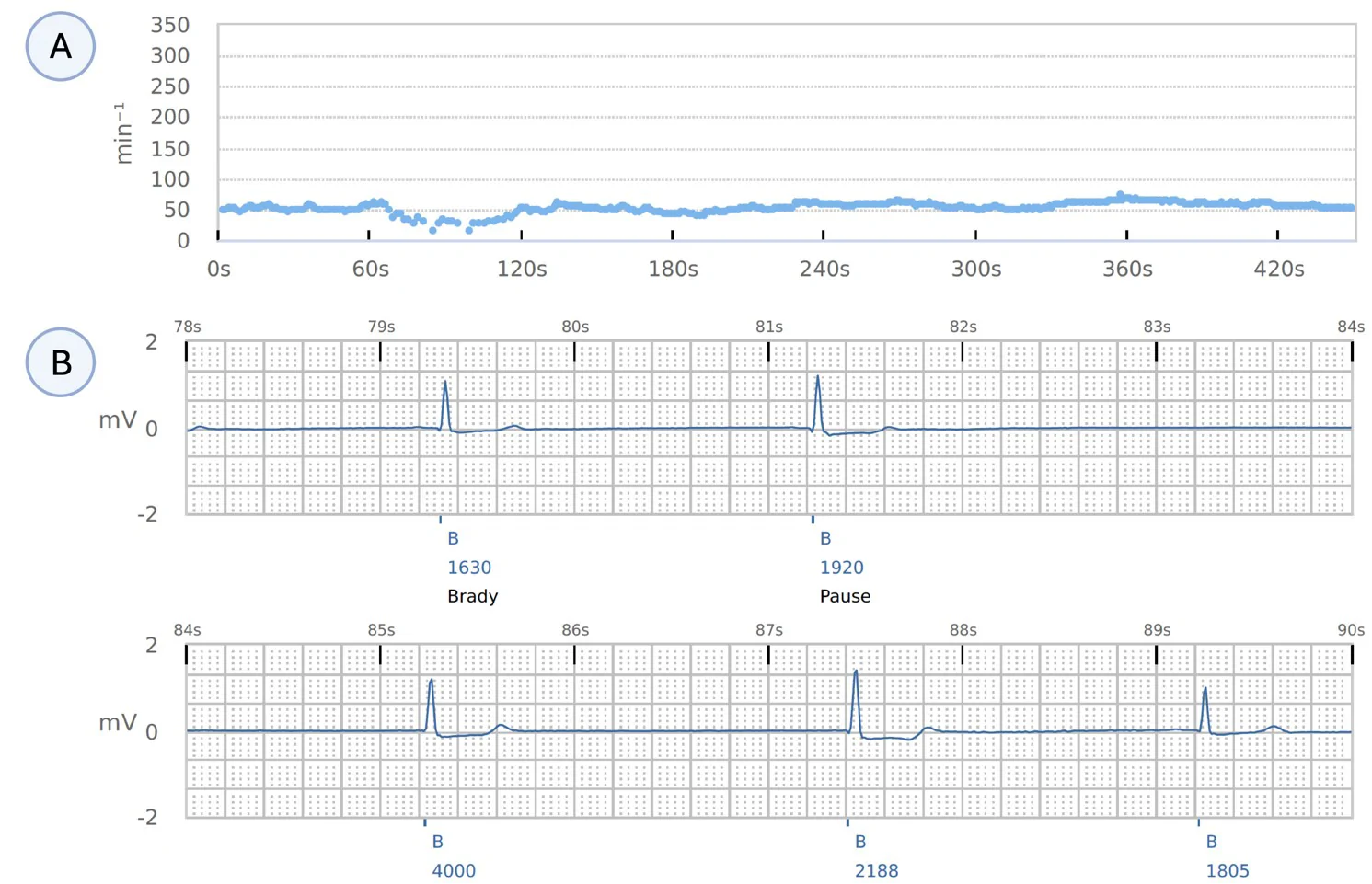
**Example of an implantable loop recorder (ILR) recording in an elderly patient with recurrent syncope**. The patient had received a LUX-Dx II+ device (Boston Scientific, Marlborough, MA, USA). The episode was patient-triggered in response to dizziness and presyncope. (A) Heart rate trend over a 450-second period. (B) Continuous ECG tracing demonstrating sinus arrest with a junctional escape rhythm and a pause exceeding four seconds. Based on these findings, the patient was contacted and promptly scheduled for pacemaker implantation. ILR, implantable loop recorder.

The benefit of ILR for AF screening in patients at increased stroke risk is rather ambiguous. The LOOP trial compared prolonged monitoring with an ILR with standard care. The AF detection rate with an ILR was higher compared to standard care (31.8 vs. 12.2%) over a median follow-up of 5.5 years. However, there was no significant difference in the occurrence of stroke or systemic arterial embolism [59]. Regarding device-detected subclinical AF, two large trials showed overall low stroke rates in these patients (NOAH-AFNET 6: ∼1%/year; Artesia: 0.64%/year with apixaban vs. 1.02%/year with aspirin) [60,61]. In the NOAH-AFNET 6 trial anticoagulation with Edoxaban did not result in a significantly reduced incidence of a composite of cardiovascular death, stroke, or systemic embolism as compared with placebo [60]. In the Artesia trial, anticoagulation with apixaban resulted in a significant reduction of ischemic strokes, especially with regards to disabling or fatal events [61]. However, in both trials, major bleeding was more frequent than ischemic events in patients receiving oral anticoagulation. Accordingly, in these patients, shared decision-making regarding the initiation of oral anticoagulation remains crucial.

## 5. Contemporary Use Cases of Short Duration Holter ECG

Despite all the available smart devices, a short-duration Holter ECG is still an inexpensive diagnostic test for patients with syncope, dizziness or palpitations occurring at least weekly. In patients whose symptoms are less frequent, diagnostic yield declines rapidly and other devices should be considered [13].

In patients with suspected vasospastic angina a 12-lead ambulatory Holter ECG should be performed according to the 2024 ESC guidelines on the management of chronic coronary syndromes (class IIaB recommendation) [62]. Furthermore, a 12-lead Holter ECG can also aid in the diagnosis of Brugada Syndrome. Gray et al. [36] showed that 34% of patients who were diagnosed by provocation testing only exhibited a type 1 Brugada pattern at least once during a 24-hour 12-lead Holter ECG. In practice, a 12-lead Holter ECG could help detect a spontaneous type 1 Brugada pattern and, if found, obviate the need for provocation testing.

## 6. Conclusion: Which Device for Which Patient?

Smartphones, wearables, ECG patches and AI-based rhythm analysis have revolutionized arrhythmia care. They nowadays enable low-threshold and widely available arrhythmia diagnostics that must be tailored according to the symptoms and their frequency of occurrence. Short-duration Holter ECGs are still a reliable method in frequently occurring symptoms while smartwatches or ECG patches are the preferred choice in patients with less frequently occurring palpitations to achieve a symptom-arrhythmia-correlation. In patients with unexplained syncope an ILR is the preferred choice due to its continuous rhythm surveillance as recommended by international guidelines. Fig. 4 presents a flowchart for ECG device selection. AF screening can be done with any of these devices, however, at the moment it is unclear which patients benefit from AF screening. It is also unknown which device would be the primary choice with regards to cost-effectiveness, as, to our knowledge, there are no studies comparing different screening strategies.

**Fig. 4.**
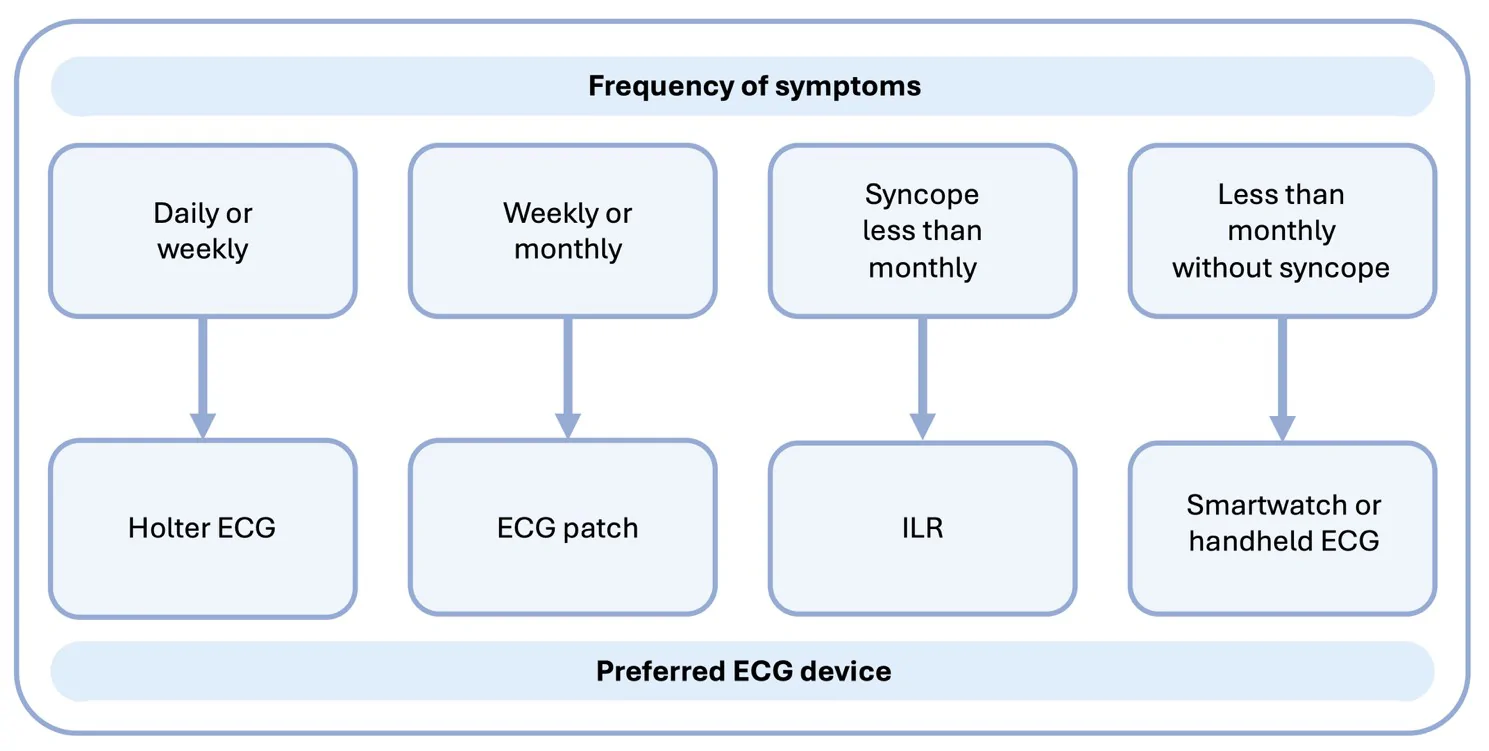
**Flowchart for ECG device selection in patients with arrhythmia symptoms**. Device selection is based on symptom frequency. In cases of rare episodes, the presence of syncope favors an ILR insertion. In the absence of syncope, a SL-ECG recorded by a smartwatch or wearable is recommended. ILR, implantable loop recorder; SL-ECG, single-lead electrocardiogram.

This review focuses on ECG recording devices for the adult population. Regarding its use in children, a recently published systematic review found that SL- and ML-ECGs recorded with wearables as well as ECG patches yielded high signal quality, thus enabling manual interpretation [63]. However, it is important to note that most of these devices are used off-label in children, and data for very young children remain scarce. There is also insufficient evidence for the use of these devices in pregnant women.

At the moment, wearables and patches have been approved for only selected indications while a standardized integration into diagnostic pathways is often missing. Their full potential will be enabled by an optimal interaction of technical precision, AI-based analysis, and clinical relevance. Further smart devices that collect metabolic data have already been released or are on the horizon. In combination with wearables for arrhythmia care, they might enable a more holistic approach in the prevention and management of cardiac diseases.

## Abbreviations

AI, artificial intelligence; AF, atrial fibrillation; ARVC, arrhythmogenic right ventricular cardiomyopathy; bpm, beats per minute; CE, Conformité Européenne; ECG, electrocardiogram; FDA, US Food and Drug Administration; HCM, hypertrophic cardiomyopathy; ILR, implantable loop recorder; ML-ECG, multi-lead electrocardiogram; PPG, photoplethysmography; PPV, positive predictive value; PSVT, paroxysmal supraventricular tachycardia; SL-ECG, single-lead electrocardiogram; TAVI, transcatheter aortic valve implantation.
